# CO_2_ Capture and Gas Storage Capacities Enhancement of HKUST-1 by Hybridization with Functionalized Graphene-like Materials

**DOI:** 10.1021/acs.energyfuels.2c04289

**Published:** 2023-03-15

**Authors:** Valentina Gargiulo, Alfonso Policicchio, Luciana Lisi, Michela Alfe

**Affiliations:** † CNR-STEMS Institute of Sciences and Technologies for Sustainable Energy and Mobility, P. le V. Tecchio 80, Napoli 80125, Italy; ‡ Dipartimento di Fisica, Università della Calabria, Via P. Bucci - Cubo 31C, Arcavacata di Rende 87036, Italy; § CNISM - Consorzio Nazionale Interuniversitario per le Scienze fisiche della Materia, Via della Vasca Navale 84, Roma 00146, Italy; ∥ Consiglio Nazionale delle Ricerche, Istituto di Nanotecnologia (Nanotec) − UoS Cosenza, Via Ponte P. Bucci, Cubo 31C, Arcavacata di Rende 87036, Italy

## Abstract

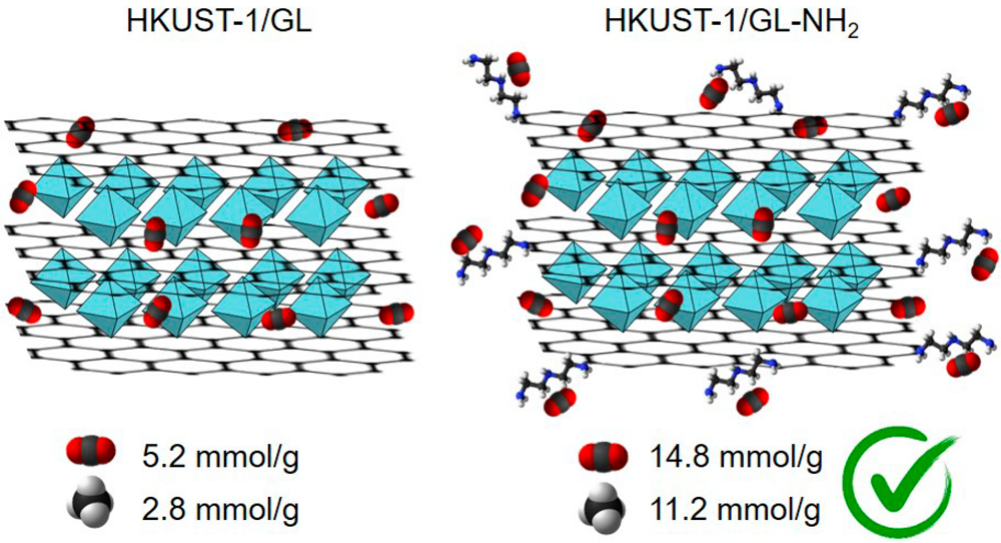

The role of graphene related material (GRM) functionalization on the structural and adsorption properties of MOF-based hybrids was deepened by exploring the use of three GRMs obtained from the chemical demolition of a nanostructured carbon black. Oxidized graphene-like (GL-ox), hydrazine reduced graphene-like (GL), and amine-grafted graphene-like (GL-NH_2_) materials have been used for the preparation of Cu-HKUST-1 based hybrids. After a full structural characterization, the hybrid materials underwent many adsorption–desorption cycles to evaluate their capacities to capture CO_2_ and store CH_4_ at high pressure. All the MOF-based samples showed very high specific surface area (SSA) values and total pore volumes, but different pore size distributions attributed to the instauration of interactions between the MOF precursors and the specific functional groups on the GRM surface during MOF growth. All the samples showed a good affinity toward both gases (CO_2_ and CH_4_) and a comparable structural stability and integrity (possible aging was excluded). The trend of the maximum storage capacity values of the four MOF samples toward CO_2_ and CH_4_ was HKUST-1/GL-NH_2_ > HKUST-1 > HKUST-1/GL-ox > HKUST-1/GL. Overall, the measured CO_2_ and CH_4_ uptakes were in line with or higher than those already reported in the open literature for Cu-HKUST-1 based hybrids evaluated in similar conditions.

## Introduction

The Materials 2030 Manifesto, codeveloped and cosigned on February 2022 by high-level representatives from European research institutes and industries, stressed the importance of the EU’s technology leadership and strategic autonomy and the role of advanced materials.^1^ In the document, advanced materials are identified as key enabling technologies to deliver solutions to reach climate neutrality, circularity, and sustainability. In particular, in the document, advanced materials are recognized as key to providing solutions for many applications, including those dealing with carbon capture, utilization, and storage technologies (CCS and CCUS).^1^

CCS groups the techniques focused on CO_2_ capture from stationary point sources or directly from the atmosphere and subsequent permanent sequestration in deep underground geologic formations (storage). Different types of CO_2_ sources can be managed by selecting between three types of capture approaches: precombustion, postcombustion, and oxygen-enriched combustion.^2^

It is widely assessed that the cost associated with the capture step is one of the largest challenges that hinder CCUS large-scale deployment.^2^ In this scenario, the EU Technology Development Report^3^ identifies the following as the main challenges for the development of carbon capture options involving materials: (i) materials optimization for severe conditions (high temperature, presence of humidity, and impurities); (ii) increased material availability and reduced costs for material production and plant assembling; (iii) standardized testing procedures.

Metal organic frameworks (MOFs) are emerging as high-capacity adsorbents for CO_2_, as testified by the different review papers available in the literature.^4−6^ MOFs are a class of solid porous materials classified as a subclass of coordination networks, which are a subclass of coordination polymers.^7^ Their 3D structure arises from the self-assembling of metal nodes (metal ions, metal centers, or metal clusters) and organic linkers through the instauration of strong coordination bonds. Their distinctive structural features are high porosity, large volume of the pores, and good thermal stability (250–500 °C).^8^ MOFs are promising materials for environmental and biomedical applications, they have been used as catalysts, absorbents for toxic gases and metal ions, materials for electrochemical device preparation, proton-conducting materials, drug carriers, bioimaging agents, and therapeutic agents^9−16^ and as novel sensing materials.^17^ As a drawback, most of the MOFs suffer poor thermal, chemical, and mechanical stability under extreme conditions and exhibit a very low electrical conductivity and poor electrocatalytic ability.^18−21^

To expand the potential use of MOFs, also in large-scale applications, most of the above must be addressed, boosting the research focused on MOFs designing and new approaches to MOF-based composites and hybrids. The production of MOF-based composites and hybrids is an attractive approach since the combination of MOFs with suitable materials can improve the overall functionality, porosity, synthetic conditions, and thermal/magnetic/electric properties to meet specific requirements. Polymers,^22^ metal oxides,^23^ nanoparticles,^24^ carbon nanotubes,^25^ quantum dots,^26^ and graphene related materials (GRMs)^27^ are the most common materials used for the preparation of MOF composites and hybrids.

MOF/GRM composites are particularly promising due to the possibility to achieve synergic effects between the MOF (e.g., controlled porosity, selectivity, catalytic activity) and GRMs (e.g., ionic and electric conductivity, light absorption, mechanical stability). Thanks to the presence of heteroatoms containing functional groups and the aromatic sp^2^ domains, GRMs can not only act as fillers but also participate in bonding interactions, enhancing the coordination bonding and influence the growth of MOF.^28−30^ Among the different components of the GRMs family, graphene (G), graphite oxide, graphene oxide (GO), and reduced graphene oxide (rGO) are the most used materials. The use of GRM decorated with specific functional groups (e.g., N-containing groups) for the preparation of MOF composites is also an attractive option but is currently poorly investigated.^31−33^

In most cases, the MOF composites featured better thermal stability in comparison to the parent MOF. The intercalation with GRM also provides for electrical conductivity, while the pristine MOF is usually insulating.^34^ Depending on the structure of the MOF, the interactions between metal nodes and the GRM can lead to an increase of defects and micropore volume of the composites, resulting in a higher concentration of unsaturated metal centers that serve as adsorption sites for gases.^35^

In this work, three GRMs obtained from a nanostructured carbon black (CB) (oxidized graphene-like (GL-ox), hydrazine reduced graphene-like (GL), and amine-grafted graphene like materials (GL-NH_2_)) have been used for the preparation of HKUST-1 hybrids. The obtained materials have been characterized in terms of chemico-physical and morphological features, and their capacities to capture CO_2_ and store CH_4_ at high pressure and room temperature (RT ≅ 23 °C) have been evaluated by volumetric adsorption tests with the aim of highlighting the role of GRM functionalization on structural and adsorption MOF properties and the suitability of MOF/GRM hybrids for applications like biogas upgrading.^36−39^

## Experimental Section

Chemicals and solvents were purchased from Merck KGaA, Darmstadt, Germany (ACS grade) and used as received. The carbon black (CB) used for the GRMs production (furnace type, CB N110, according to the ASTM classification) was kindly provided by Sid Richardson Carbon Co. (Fort Worth, United States).

### Materials Synthesis

GL-ox synthesis: GL-ox was produced through a top-down approach detailed elsewhere^40,41^ and briefly summarized here: 500 mg of CB powder was oxidized with 10 mL of HNO_3_ (67%) at 100 °C under stirring and reflux for 90 h. The oxidized carbonaceous material (GL-ox) was recovered by centrifugation, washed with distilled water three times, dried at 105 °C, and stored.

GL synthesis: GL materials were synthesized accordingly with the procedure described in refs (40 and 41) and briefly summarized here: An amount of GL-ox was suspended in distilled water (1 mg/mL) and treated with hydrazine hydrate (35 μL of hydrazine for each mg of GL-ox) and the corresponding mixture was maintained at 100 °C under stirring and reflux for 24 h. After cooling down the suspension, the excess of hydrazine was neutralized with a diluted nitric acid solution (4 M) and the GL recovered as a black solid by centrifugation. GL was washed with distilled water two times and stored as water suspension (pH 3.5). The suspension keeps stable over time.

GL-NH_2_ synthesis: 200 mg of GL-ox was suspended in 80 mL of ethanol at a mass concentration of 2.5 mg/mL, sonicated for 20 min, and then treated with 40 mL of diethyltriamine (DETA). The mixture was kept at 80 °C under stirring and reflux for 24 h. After cooling down, the suspension was filtered (Durapore Membrane Filter, 0.22 μm, Millipore) under vacuum, and GL-NH_2_ was recovered as a black solid on the filter. Several ethanol washings were performed to remove any traces of DETA. GL-NH_2_ was recovered, suspended in water and stored. The GL-NH_2_ water suspension remains stable over time.

HKUST-1 synthesis: The Cu-based MOF (HKUST-1) was synthesized in accordance with the synthetic procedure previously described^27^ and reported here in brief: 1 g of Cu(NO_3_)_2_·2.5 H_2_O and 0.5 g of benzene-1,3,5-tricarboxylic acid (BTC) were mixed with 8.5 mL of N,N-dimethylformamide (DMF) in a round-bottom flask, and the mixture was sonicated for 5 min. After that, ethanol was added (8.5 mL) and the mixture was sonicated for 5 min more, deionized water was added (8.5 mL), and finally, the mixture was sonicated for 2 h, allowing the complete dissolution of BTC crystals. Then the reaction mixture was kept at 85 °C for 21 h under stirring (solvothermal conditions). After the suspension was cooled down, HKUST-1 crystals were recovered by under-vacuum filtration and purified by several washings with ethanol and then with dichloromethane (DCM). After that, the MOF crystals were immersed in DCM for 3 days (the solvent was renewed three times) and finally recovered by filtration. HKUST-1 crystals were dried and then activated under-vacuum at 120 °C for 24 h to remove residual solvent molecules trapped in the porous network and/or coordinated to the copper centers. After activation, the HKUST-1 crystals turn from turquoise to dark blue.

HKUST-1 based hybrids synthesis: The three hybrids (HKUST-1/GL, HKUST-1/GL-ox, and HKUST-1/GL-NH_2_) were synthesized adapting the synthetic strategy described in.^27,34^ In all the three cases, the reaction conditions and the further workup were the same as those described above for the pristine HKUST-1. HKUST-1/GL-ox was synthesized by using the following amounts of precursors: 1 g of Cu(NO_3_)_2_·2.5 H_2_O, 0.5 g of BTC, and 35 mg of GL-ox. HKUST-1/GL was synthesized by using these amounts of precursors: 1 g of Cu(NO_3_)_2_·2.5 H_2_O, 0.5 g of BTC, and 35 mg of GL materials in 8.5 mL of water. HKUST-1/GL-NH_2_ was synthesized with the following amounts of precursors: 1 g of Cu(NO_3_)_2_·2.5 H_2_O, 0.5 g of BTC, and 35 mg of GL-NH_2_ in 8.5 mL of water. Both GL and GL-NH_2_ were added to the reaction mixture as a water suspension after the addition of DMF and ethanol, replacing the pure water volume used in the preparation of the pristine HKUST-1 (8.5 mL).

### Materials Characterization Methods

The elemental composition of the materials was estimated using a CHN 628 LECO elemental analyzer. Each measurement was repeated three times. Ethylenediaminetetraacetic acid (EDTA) was used for instrument calibration. The C, H, and N contents were expressed on a weight percentage basis. The content of copper was estimated by inductively coupled plasma-mass spectrometry (ICP-MS) after a microwave-assisted acidic treatment of the MOF-based materials as reported in.^34^

The materials thermal stability was evaluated by a thermogravimetric analysis (TGA) under an inert atmosphere (N_2_, 40 mL/min) from 50 °C up to 800 °C at a rate of 10 °C/min by using a PerkinElmer STA 6000. 5–10 mg of each material were loaded in an alumina crucible for each measurement. The crucible was preconditioned at 920 °C to guarantee an accurate solid residue determination.

The specific surface area and the pore size distribution (PSD) of the MOF samples were determined from the N_2_ adsorption isotherms collected at −196 °C with a Quantachrome Autosorb 1-C after outgassing the materials under vacuum at 120 °C for 12 h. The specific surface area (SSA) was calculated by using the Brunauer-Emmet-Teller (BET) equation, while the total pore volume (*V*_tot_) was calculated from the amount of N_2_ adsorbed (expressed in cm^3^/g at STP) at a relative pressure of *p*/*p*^0^ ∼ 0.99. The PSD and the micropore volume were evaluated applying the NLDFT method and using the standard slit-pore model for N_2_ adsorption at −196 °C on carbon, which provided the best fitting of isotherm curves (fitting error 1–2%) and it is generally applied to carbon material adsorption isotherms.^42^ Due to the complete overlapping of adsorption and desorption branches, only adsorption data were used for NLDFT model fitting.

The MOF sample crystallinity was evaluated by X-ray diffraction (XRD). The measurements were performed on a PANalytical diffractometer (Malvern, Worcestershire, United Kingdom) operating with a Ni filter and a Cu Kα radiation (λ = 1.54056 Å) in the 5–80° 2θ range, with a step size of 0.02° and a counting time of 80 s per step.

FTIR spectra in the 450–4000 cm^–1^ range were acquired with a resolution of 2 cm^-1^ (8 scans for each spectrum) on a PerkinElmer MIR spectrophotometer in transmission mode. The spectra were acquired on KBr pellets and the background noise was corrected.

The morphology of the samples was evaluated by scanning electron microscopy (SEM) using a FEI Inspect S50 scanning electron microscope equipped with an EDS Oxford AZtecLiveLite probe and Xplore 30 detector for elemental analysis. Before analysis, the powdered samples were dried and sputter-coated with a thin layer of gold to avoid charging phenomena.

### Experimental Apparatus and Procedure for Gas Adsorption/Desorption Measurements

The CO_2_ and CH_4_ adsorption/desorption measurements were carried out around room temperature (RT ≅ 23 °C) between 0–15 bar and 0–50 bar, respectively, on an optimized Sievert-type (volumetric) apparatus f-PcT suited for accurate and reliable gas adsorption measurements.^43^ All the MOF samples (∼ 300 mg each) before adsorption/desorption measurements were outgassed under vacuum (10^–6^ mbar) at 120 °C overnight. On each sample, several measurements and cycles (with and without thermal treatment in between) were carried out to test the gas adsorption reproducibility.

The skeletal density of the materials^44,45^ was evaluated by helium pycnometry performed on the same experimental apparatus reported above. The measurements were performed at RT and in the pressure range of 0–0.9 bar before and after the CO_2_ and CH_4_ adsorption/desorption tests to check possible changes induced in the analyzed sample by exposure to different gas specimens and to high pressure. All pycnometry measurements were repeated at least 30–40 times to minimize the experimental error.

Adsorption and desorption isotherms were reported in terms of wt % vs *P*_eq_ where wt % = *g*_gas_/*g*_material_ × 100. Data were analyzed by means of the Töth model,^46^ which allows us to evaluate the strength of the interaction that occurs between the surface of the adsorbent and the adsorbed gas. The Töth model is an extension of the Langmuir model and it allows for obtaining information regarding the maximum storage capacities, the molecule–surface interaction, and system heterogeneity/homogeneity. In the Töth equation (eq 1)
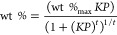
1wt %_max_ is the asymptotic maximum storage capacity, *K* is the equilibrium constant (determined by the energetic interaction between the adsorbent and the adsorbate), and *t* is a parameter introduced by Töth in order to consider the homogeneity grade of the sample surface. The smaller the parameter, the more heterogeneous the system. When *t* is equal to 1, the Töth isotherm reduces to the Langmuir isotherm, which is related to a homogeneous system.

## Results and Discussion

The elemental compositions of the three GRMs used for the preparation of MOF hybrids and of all MOF samples are reported in Table 1. The carbon content percentage for all GRMs is above 55 wt %, the hydrogen content is low (∼1 wt %) for the oxidized material (GL-ox) but very high (∼11.5 wt %) in the case of GL-NH_2_ as consequence of the presence of DETA. The oxygen content is lower in both GL and GL-NH_2_ compared with GL-ox, as a consequence of the reaction with hydrazine (e.g., formation of hydrazones) and with DETA (formation of amidic bonds). The content of nitrogen is quite high (above 4 wt %) in both GL and GL-NH_2_, testifying the formation of hydrazones and amidic bonds, respectively, while it is absent in the case of GL-ox.

**Table 1:** Materials Composition

Sample	C (wt %)	O (wt %)^b^	H (wt %)	N (wt %)	Cu (wt %)
GL-ox	59.01	40.03	0.96	-	-
GL^a^	52.9	39.7	1.40	6.09	-
GL-NH_2_	57.96	25.91	11.46	4.67	-
HKUST-1^a^	36.62	46.17	0.98	0.93	15.30
HKUST-1/GL-ox	38.83	34.92	0.95	0.52	24.78
HKUST-1/GL^a^	37.70	42.36	1.11	1.24	17.20
HKUST-1/GL-NH_2_	36.59	38.63	0.88	0.40	23.5

a From ref (27).

b O wt % content was calculated by difference.

The evidence of the presence of -NH_2_ functionalities is testified by the prominent band between 3500 and 3350 cm^–1^ in the GL-NH_2_ FTIR spectrum (see Figure S1), emerging from the broad band in the 3000–3700 cm^–1^ range related to O–H stretching vibrations (also due to possible adsorbed H_2_O) evidenced in both GL and GL-ox spectra. The absorption band around 1620 cm^–1^ related to the N–H bending mode is not clearly discernible, as it is submerged by the skeletal vibrations of the sp^2^ graphitic domains.

As concerns the MOF samples, the carbon content is between 36.5 and 39 wt % in accordance with previous literature reports,^34^ while the contents of hydrogen and nitrogen are around 1 wt %. The content of Cu, estimated by ICP-MS analysis, is between 15 and 25 wt % for all MOF samples, in agreement with previous findings.^27,34^

**Figure 1:**
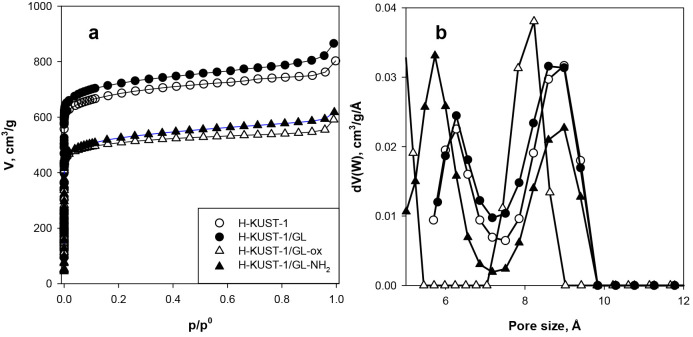
(a) N_2_ adsorption isotherms at −196 °C and (b) PSD curves of the MOF samples.

The textural properties of the MOF samples have been evaluated from the N_2_ adsorption/desorption isotherms at −196 °C. In the left panel of Figure 1, the N_2_ adsorption isotherms at −196 °C of the four MOF samples are compared; for all the samples, the desorption branch is not shown because it completely overlaps the adsorption branch, indicating the total absence of mesopores responsible for the typical hysteresis due to N_2_ capillary condensation.^47^ All isotherms show the Type I curve shape with a well-defined plateau, suggesting a small contribution of the external surface area to the adsorption. This isotherm shape is typical of microporous materials,^48^ and it is in accordance with previous reports on the textural properties of Cu based HKUST-1 MOF structure.^27,33,34,49^ A magnification of Figure 1a in the low-pressure range is reported in Figure S2 of the Supporting Information section to highlight the absence of differences in the adsorption behavior at low-pressure values among all the investigated MOF-based materials.

In Table 2 the values of specific surface area, the total pore volume, and the micropores volume of the MOF samples, evaluated as described in the section 2, are reported.

**Table 2:** Materials Textural Characteristics

Sample	Skeletal density (g cm^–3^)	Specific surface area (m^2^ g^–1^)	Micropore volume (cm^3^ g^–1^)	Mesopore volume (cm^3^ g^–1^)	Total pore volume (cm^3^ g^–1^)	*V*_mic_/*V*_T_ (%)
HKUST-1^az^	2.32 ± 0.05	2632	0.987	0.123	1.11	89
HKUST-1/GL-ox	2.29 ± 0.04	1970	0.826	0.074	0.90	92
HKUST-1/GL^az^	1.45 ± 0.05	2768	1.026	0.174	1.20	86
HKUST-1/GL-NH_2_	2.44 ± 0.03	2010	0.744	0.106	0.85	88

az From ref (27).

As clearly visible, all samples show very high specific surface area and total pore volume values. In all the cases, the micropore volume is between 86 and 92% of the total pore volume (see Table 2) indicating a very low contribution coming from the mesopores.

The effect of the type of GRMs used to intercalate the HKUST-1 structure on the hybrid textural properties can be summarized as follows: the use of GL leads to a slight increment of the SSA and total pore volume in agreement with previous findings,^27^ while the use of GL-NH_2_ and GL-ox leads to a worsening (see Table 2). This result meets some previous literature findings, where the intercalation of GRMs in MOF structures corresponds to a decrease of both SSA and total pore volume^27^ confirming that the effect of the intercalation of graphenic material into a MOF structure is also dependent on the specific GRM adopted.

The pores size distribution (PSD) curves reported in Figure 1b clearly show that all samples contain mainly small micropores (<10 Å) which are responsible for the very high values of surface area. Figure 1b evidenced that the pristine HKUST-1 presents a bimodal distribution of micropores centered at about 6 and 9 Å, respectively, which is almost preserved upon GL intercalation. The intercalation with GL-ox leads to a quite different PSD characterized by a single peak centered at 8 Å and an arising peak below 6 Å ascribable to narrower micropores. It is noteworthy that for HKUST-1/GL-ox an underestimation of micropore volume and, as consequence, of the SSA should be supposed since narrower micropores are not well detected by N_2_ physisorption because of the limited diffusion of N_2_ molecules at −196 °C in small sized pores.^47^ As concerns the effect of the intercalation with GL-NH_2_ on the PSD of the hybrid material, a reduction of the volume of larger micropores with diameters centered around 9 Å and an increase of the volume of small micropores shifted toward smaller sizes are detected.

The different effects exerted by the three GRMs on the textural properties can be ascribed to the instauration of interactions between the MOF precursors (namely, the carboxylic functional groups and/or the unsaturated copper ions) and the specific functional groups on the GRMs surface. GL-ox is very rich in oxygen containing functional groups, mainly carboxylic groups,^53^ prone to interact with the Cu (II) ions generating HKUST-1 nucleation sites anchored to the GRM surface. This is in agreement with the hard–soft-acid–base theory,^54^ a useful tool to interpret the adsorption of metal ions onto activated carbons where soft ions are better adsorbed by carbon, nitrogen, sulfur, or chloride containing surface groups, while hard ions (as Cu(II)) are preferentially adsorbed by oxygenated or fluorinated groups. This phenomenon can be responsible for an alteration in the MOF structure growth and of the remarkable changes in the PSD of the HKUST-1/GL-ox compared to the pristine HKUST-1. In the case of GL^41^ and GL -NH_2_, the low content of carboxylic groups and the presence of nitrogen containing functional groups limit the instauration of such interactions and thus the PSDs of the corresponding hybrids resemble that of the pristine HKUST-1.

In Table 2, the skeletal density values of the MOFs materials evaluated through He pycnometry are also reported. The intercalation with GL-ox and GL-NH_2_ caused only slight changes in the skeletal density of two of the hybrid materials with respect to the parent MOF, while the intercalation with GL induces a larger change in the skeletal density, consistent with the higher surface area detected. The comparison of the skeletal density values before and after the whole adsorption cycles (before and after CO_2_ and CH_4_ adsorption cycles up to 15 and 50 bar, respectively) confirms that the adsorption process performed at high pressure induces a negligible pore collapse (around 5%).

**Figure 2:**
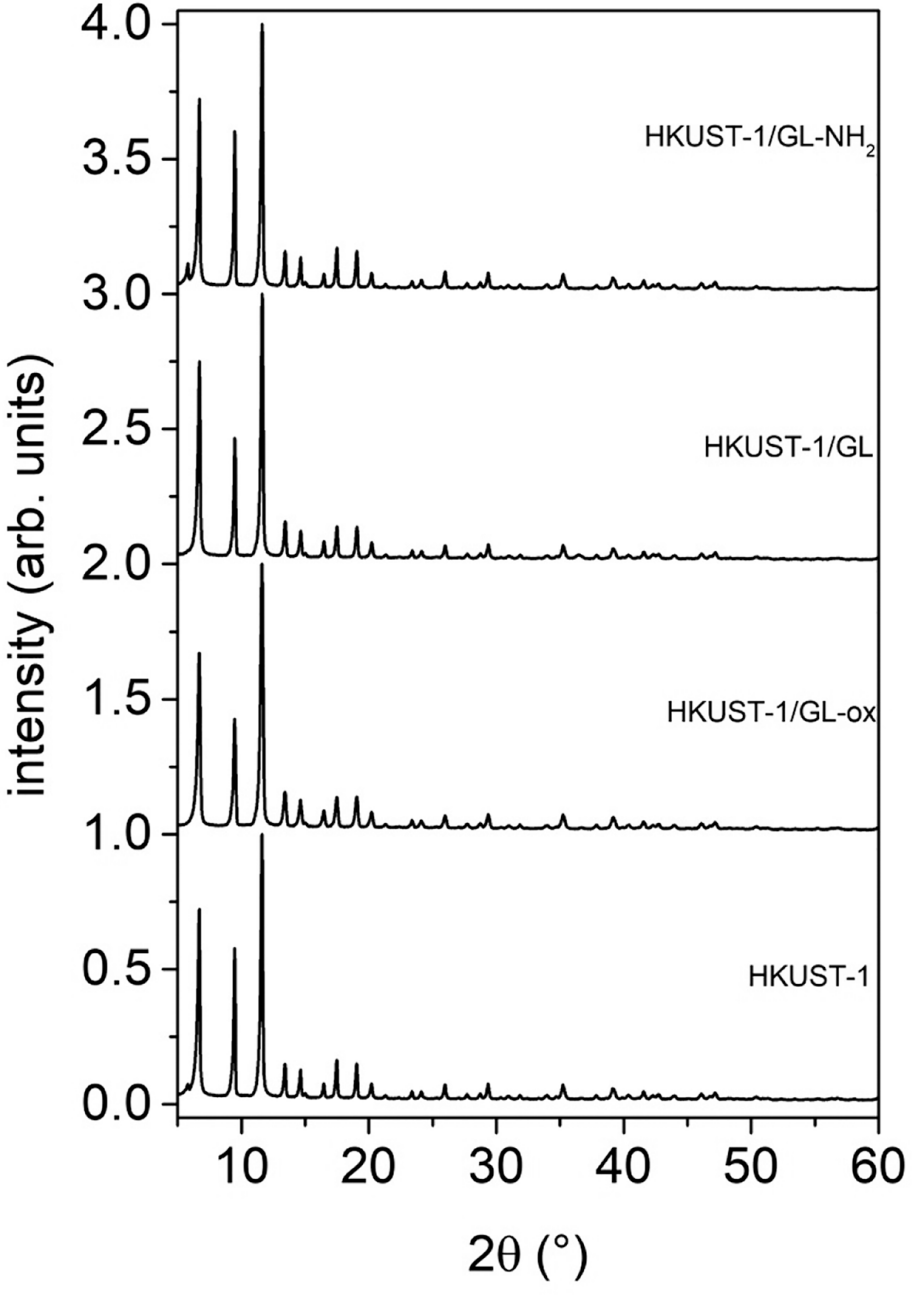
XRD diffraction patterns (height normalized and shifted for clarity) of HKUST-1 and the three hybrids.

The crystallinity of the samples was probed by X-ray diffraction. The diffraction patterns of the MOF samples are shown in Figure 2.

The XRD patterns of all the MOF samples are very similar, containing intense and sharp peaks located below 2θ = 15°. In particular, the three main peaks are located at 6.7, 9.4, and 11.6° and correspond to the diffraction peaks of the 200, 220, and 222 octahedral crystal planes.^55^ The pristine HKUST-1 shows an XRD pattern that perfectly agrees with the one reported in the literature for the same MOF structure,^49−52,55^ and it is the result of the typical octahedral shape of HKUST-1 crystals. The three hybrids, exhibiting a pattern similar to that of the pristine MOF, are characterized by the same crystal shape, confirming that the presence of GRMs in the reaction mixture does not negatively influence the MOF crystal growth.^27,34,49−52^

**Figure 3:**
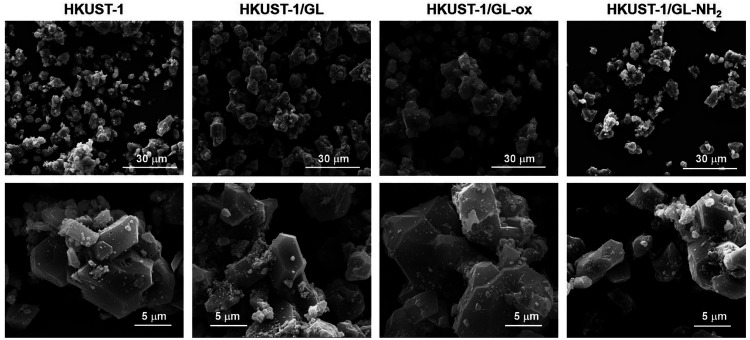
SEM images of MOF samples at different magnifications (3000×, top; 12 000×, bottom).

The octahedral shape of the crystals of all of the MOF samples was also confirmed by SEM imaging. In Figure 3, SEM images at different magnifications of the MOF samples are reported.

**Figure 4:**
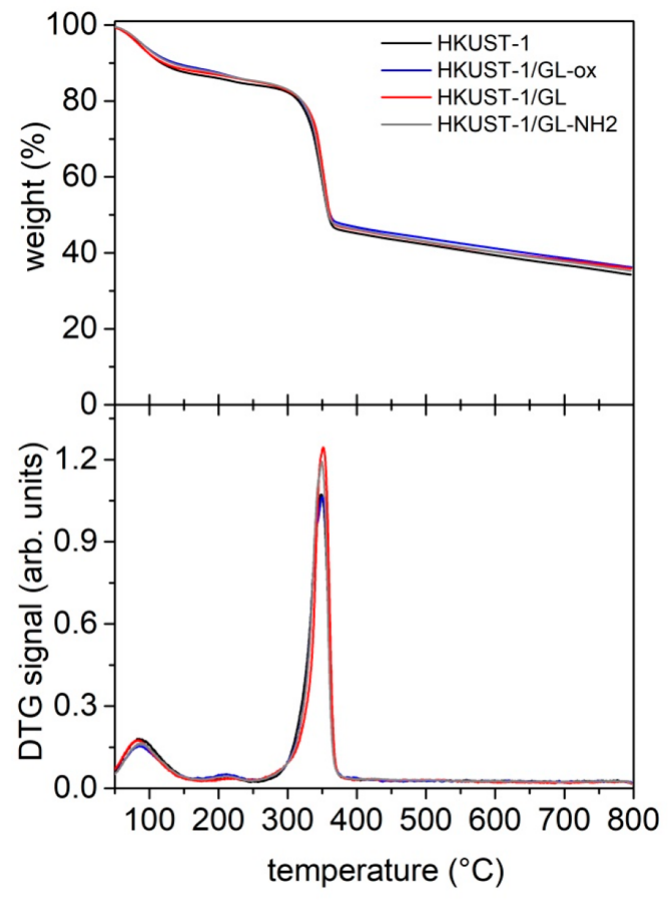
TG (top) and derivative TG (DTG) (bottom) profiles of the MOF samples.

Thermogravimetric analysis (TGA) indicated, overall, a thermal stability up to 300 °C for all of the MOF samples (see Figure 4).

The TG curves of all analyzed samples are very similar, exhibiting a first weight loss around 100 °C ascribable to moisture removal and a significant weight loss around 300 °C due to the decomposition of linker units leading to the collapse of the framework. For all the solids a residue around 35–40 wt % is detected, in agreement with previous findings.^34^

**Figure 5:**
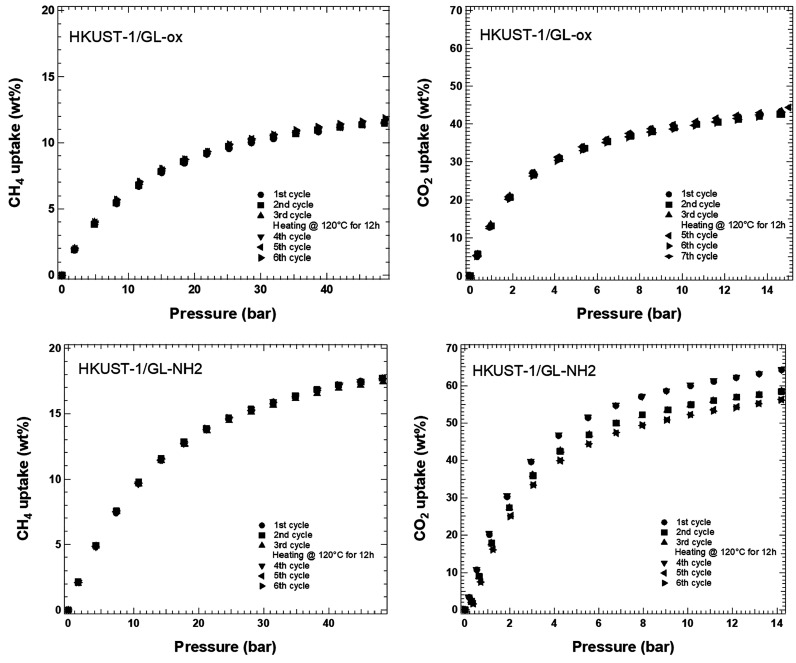
CH_4_ adsorption isotherms up to 50 bar and CO_2_ adsorption isotherms up to 15 bar were recorded during six cycles at RT. The magnitude of the error is the symbol itself.

Figure 5 shows the CH_4_ and CO_2_ adsorption isotherms of HKUST-1/GL-ox and HKUST-1/GL-NH_2_. For the sake of completeness, the adsorption isotherms of pristine HKUST-1 and HKUST-1/GL, already reported in ref (27), are shown as Figure S3 after reshaping the graphics (units of measurement conversion and *y* axis rescaling) for an easy comparison.

The adsorption process is favorable for the two hybrids and for both gases (CH_4_ and CO_2_), as testified by the concave shape of the curves. To consolidate this behavior and to establish the occurrence of a possible passivation of the adsorption sites or poisoning of the samples after subsequent gas exposure, the samples underwent subsequent adsorption/desorption cycles with and without a thermal treatment in between. The trends reported in Figure 5 indicated that both samples showed a good affinity toward both gases and a comparable structural stability and integrity (no aging occurs). Looking at the adsorption and the desorption curves reported in Figure S4 (see the Supporting Information) and taking into account the slowness of the release process (adsorbed molecules take time to be released just by pressure differences when void is not applied, as in this case), a quite total absence of hysteresis for most of the samples can be assumed and the adsorption process can be considered completely reversible. The good overlap between the adsorption and the desorption curves indicated also that the nature of the interaction between the samples and the gas molecule was mostly a physisorption process. A little deviation from this behavior was highlighted only for HKUST-1/GL-NH_2_ toward CO_2_, indeed the maximum adsorption uptake decreases by approximately 10 wt % after the first run, and the adsorption isotherms subsequent to the second cycle appear quite stable within the experimental error. Since the adsorption capacity of the samples can be easily recovered after the thermal treatment (heating up to 120 °C overnight), it can be concluded that the aging of the HKUST-1/GL-NH_2_ sample is limited to the first cycles after the activation and it is full reversible. This behavior can be explained hypothesizing, during the first adsorption cycle, the introduction of stronger interactions between the CO_2_ and the adsorbent structure (chemisorption) concurrent to the physisorption process. This behavior has been reported also by other authors for hybrids with aminated GO.^31−33,56^ Unfortunately, due to instrumental limitations, the expected hysteresis in the adsorption/desorption isotherms of HKUST-1/GL-NH_2_ that can be ascribed to the chemisorption behavior is not detectable.

**Figure 6:**
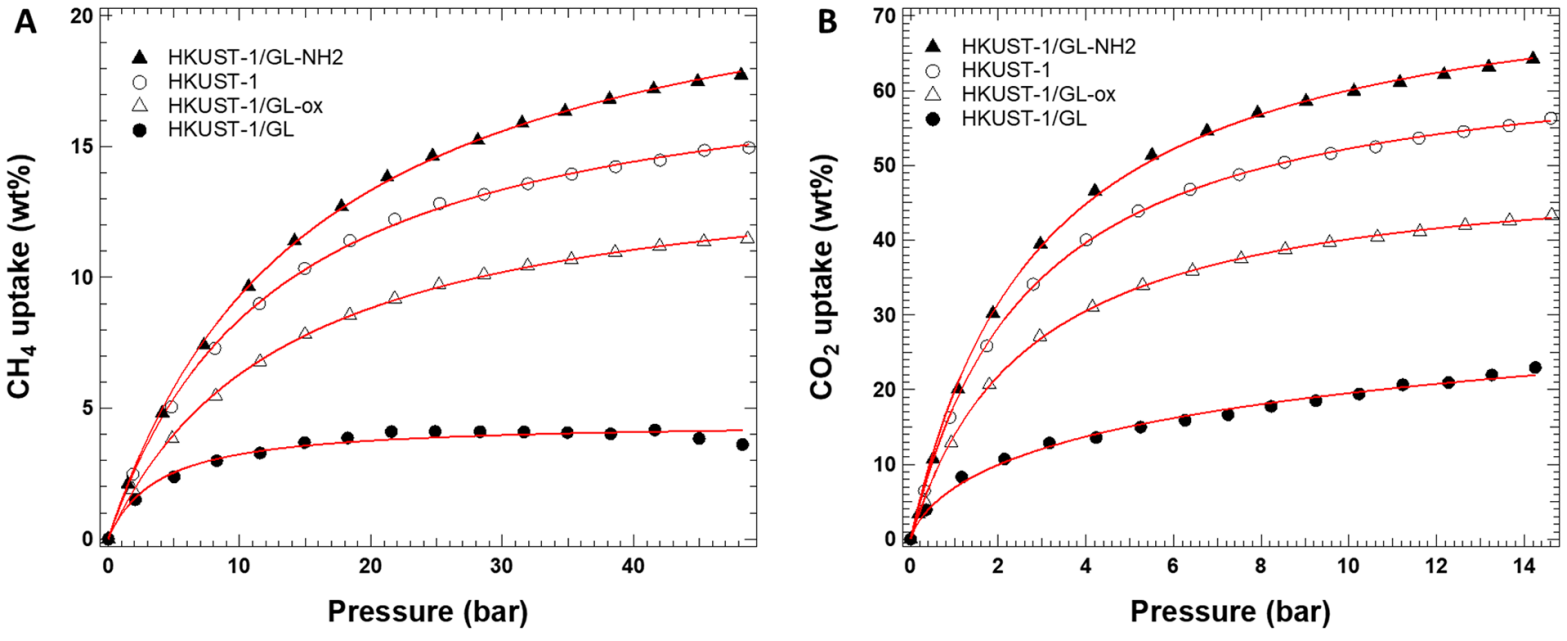
Fitted adsorption curves of each MOF sample toward CH_4_ and CO_2_. Solid lines represent fits to the Töth equation.

In Figure 6, the fitted adsorption curves of all MOF samples toward CH_4_ and CO_2_ are contrasted. Töth model^46^ was used to fit the experimental data with a good accordance.

The maximum storage capacity values of the investigated MOF samples were compared with those of the parent MOF (HKUST-1) and the hybrid with GL (HKUST-1/GL) in Table 3 and Figure 6. The trend of the adsorption capacities toward CH_4_ and CO_2_ is HKUST-1/GL-NH_2_ > HKUST-1 > HKUST-1/GL-ox > HKUST-1/GL. For both gases, the best adsorption performances are obtained with HKUST-1/GL-NH_2_ and the worst with HKUST-1/GL (see Figure 6).

**Table 3:** Adsorption Results and Comparison with Literature Data (Volumetric Apparatus, Temperature Range 20–35 °C, Pressure Range 0–15 bar for CO_2_ and 0–50 bar for CH_4_)

Gas	Material	% GRM	Synthetic method	SSA (m^2^/g)	Adsorption test methodology	Adsorption results (mmol/g)	Ref
CO_2_	HKUST-1/GL-ox	5	hydrothermal	1970	Apparatus: static volumetric system; Conditions: CO_2_, 23 °C and 0–50 bar	9.8	This work
	HKUST-1/GL-NH_2_	5		2010		14.8	
	HKUST-1	0	hydrothermal	2632	Apparatus: static volumetric system; Conditions: CO_2_, 25 °C and 0–50 bar	12.8	(27)
	HKUST-1/GL	5		2768		5.2	
	HKUST-1	0	hydrothermal	1048	Apparatus: static volumetric system; Conditions: CO_2_, 32 °C and 5 atm	1.8	(56)
	HKUST-1/GO	10		1015		2.5	
	HKUST-1	0	hydrothermal	892	Apparatus: static volumetric system; Conditions: CO_2_, 25 °C and 0–1.5 bar	7.09	(57)
	HKUST-1/GO	10		1010		8.98	
	HKUST-1/GO-Urea1	10		864		6.75	
	HKUST-1/GO-Urea2	10		936		9.16	
	HKUST-1/GO-Urea3	10		1367		13.41	
	HKUST-1		room-temperature ultrafast synthesis under ultrasound	1760	Apparatus: static volumetric system; Conditions: CO_2_ 25 °C and 1 bar	5.33	(58)
	HKUST-1/GO	2		1820		5.12	
	HKUST-1/GO	5		1520		4.79	
	HKUST-1/GO	10		1380		4.11	
	HKUST-1		mixed solvent method	1580	Apparatus: static volumetric system; Conditions: CO_2_ 30 °C and 1 bar	∼2	(52)
	HKUST-1/GO	1%		1772		∼2.5	
	HKUST-1		hydrothermal	1379.87	Apparatus: static volumetric system; Conditions: CO_2_ 25 °C and 1 bar	3.55	(49)
	HKUST-1/GO			1096.46		2.53	
	HKUST-1/UV-GO	1%		1005.63		4.07	
	HKUST-1/UV-GO	10%		1323.49		5.14	
	HKUST-1		hydrothermal	1193	Apparatus: static volumetric system; Conditions: CO_2_ 25 °C and 1 bar	∼4.5	(50)
	HKUST-1/GO	2%		1554		∼5	
CH_4_	HKUST-1/GL-ox	5	hydrothermal	1970	Apparatus: static volumetric system; Conditions: CH_4_, 23 °C and 0–15 bar	7.4	This work
	HKUST-1/GL-NH2	5		2010		11.2	
	HKUST-1	0	hydrothermal	2632	Apparatus: static volumetric system; Conditions: CH_4_, 25 °C and 0–15 bar	9.5	(27)
	HKUST-1/GL	5		2768		2.8	
	HKUST-1		hydrothermal	1137	Apparatus: volumetric apparatus; Conditions: CH_4_, room temperature and 65 bar	9.7	(59)
	HKUST-1/GO	10		1259		11	
	HKUST-1/rGO	10		1271		12	
	HKUST-1		aerogel	1140	Apparatus: volumetric apparatus; Conditions: CH_4_, room temperature and 10 MPa	6.23	(60)
	HKUST-1/GO aerogel	14		1140		9.35	

Looking at Figures 5 and 6 and Figures S3 and S4, it is evident that all of the analyzed materials tend to adsorb CO_2_ preferentially than CH_4_. The different curvature of the adsorption isotherms reported in Figure 6 indicates a different speed of saturation of the adsorption sites, while HKUST-1/GL exhibited quicker saturation for both the analyzed gases. In the case of CO_2_ adsorption, none of the samples reach saturation, indicating the possibility of an uptake improvement increasing pressure and/or changing working temperature conditions. HKUST-1/GL reached saturation for CH_4_ adsorption. In addition, HKUST-1/GL is the sample exhibiting the noisier isotherms (Figures S3 and S4) and the shape of the desorption curves for both gas specimens indicates a slow diffusion of the molecules and, consequently, a slow desorption process.

The gas uptakes evaluated for two of the three hybrid materials (HKUST-1/GL and HKUST-1/GL-ox) analyzed are overall lower than those of the pristine material (HKUST-1). The lower gas uptake compared to that of the pristine MOF suggested that the intercalation with GL or its oxidized form induces changes in the properties/chemistry of the pores that limit the adsorption properties despite the promising textural properties (high surface area and high pore volumes, reported in Table 2). Better performances are obtained by intercalating the HKUST-1 structure with GL functionalized with amino groups; in the case of CO_2_ adsorption, it can be assumed that the presence of such functionalities generates a surface chemistry inside the pores more favorable to the adsorption of the analyzed gas (CO_2_ is an acidic gas). This aspect is particularly relevant at low pressure (<2 bar), indeed looking at the CO_2_ adsorption curves of HKUST-1 and HKUST-1/GL-NH_2_ reported in Figure 6 in the range 0–4 bar, the uptakes of HKUST-1/GL-NH_2_ are always superior than those of HKUST-1, while in the same pressure range, the CH_4_ adsorption curves of HKUST-1 and HKUST-1/GL-NH_2_ (Figure 6) are completely overlapped. It is well assessed that CH_4_ adsorption is not influenced by the presence of unsaturated metal centers or other specific functionalities resulting in a pure physisorption process.^57^ On the other side, the CO_2_ adsorption, being CO_2_ an acidic molecule, is influenced also by the surface chemistry of the adsorbent,^32^ so in the case of hybrid materials a combination of chemisorption and physisorption phenomena should be taken into account. The possible occurrence of a chemisorption contribution in the case of HKUST-1/GL-NH_2_ is also suggested by the need for using a mild thermal treatment to regenerate the material after the first cycle of CO_2_ adsorption/desorption (see Figure 5).

In Table 3, a list of literature available data on gas uptakes of HKUST-1/GRM hybrids estimated at high pressure in a volumetric apparatus is proposed. As already reported in our previous works, the measured CO_2_ and CH_4_ uptakes are in line and always higher than those already reported in the open literature for Cu-HKUST-1 evaluated in similar conditions (volumetric apparatus, temperature range 20–35 °C, pressure range 0–15 bar for CO_2_ and 0–50 bar for CH_4_).^27^

As concerns the CO_2_ and CH_4_ uptake values of the hybrid materials, the comparison with already published data is not so easy to perform since a great variability in the explored conditions and in the hybrid composition (type of GRM used to intercalate HKUST-1 structure) is present.

The CO_2_ adsorption estimated in this work for the hybrid material prepared intercalating HKUST-1 structure with GL-ox (HKUST-1/GL-ox) is always superior to all the values listed in Table 3 regarding HKUST-1/GO hybrids. The value estimated for the hybrid material HKUST-1/GL is comparable with the values reported by Szczęsniak et al.,^58^ Varghese et al.,^49^ and Xu et al.^50^ for HKUST-1/GO hybrids but lower with respect to the values reported by Policicchio et al.^57^ Probably the better performance exhibited by HKUST-1/GL-ox with respect to HKUST-1/GL is ascribable to the textural properties exhibited by HKUST-1/GO that fit better with the CO_2_ adsorption at high pressure.

As concerns the CH_4_ uptake, the adsorption values estimated in this work for the three hybrid materials are always lower compared to those evaluated by Rosado et al.^60^ Anyway, it must be underlined that the materials proposed by Rosado are aerogels,^60^ structures that are very different from the materials investigated here.

In Table 3, only one work on data regarding hybrids produced with aminated GRMs is listed, since only few works on this category of materials are available^31−33,56^ and also because the approaches used to measure the gas uptakes are different from that applied in this work.

Some authors have done a lot of work on the evaluation of the effects of GRM functionalization on the properties of gas adsorption exhibited by MOF-based hybrid materials, focusing their study on the effect of aminated forms of GO on the CO_2_ uptakes of MOF5 and CuBTC hybrids.^31−33^ They tested the possibility to functionalize GO with urea, ethylenediamine (EDA), and the amino acid l-arginine^31−33^ and in all the cases they found that the presence of nitrogen containing groups improved the CO_2_ uptake of the hybrid with respect to that of the pristine material. More in detail, they found that CuBTC-based materials produced by functionalizing GO with urea or l-arginine always exhibited superior CO_2_ uptakes with respect to the pristine MOF,^33^ and in the case of hybrids produced with MOF5 only at pressure lower than 1 bar, this trend was confirmed.^32^ In all the cases, the results were interpreted taking into account the textural properties as well as the introduction of specific interactions between CO_2_ molecules and reactive sites on the inner surface of pores (nitrogen containing groups and/or unsaturated metal sites). In accordance with our results, the work of Zhao et al.^31−33^ confirmed that CO_2_ is mainly physisorbed, but at the same time, they underline that also the surface chemistry plays a role since, at low pressure conditions, specific interactions could be relevant (chemisorption).

For a complete characterization of the adsorption properties of the hybrid materials, the application of the Töth model equation to calculate wt %_max_ values for both CH_4_ and CO_2_ adsorptions was performed (values are listed in Table 4). Starting from those values, in accordance with the following equation (eq 2), the selectivity (*S*) of each MOF sample for a simulated 50:50 CO_2_ and CH_4_ mixture (as representative of conditions encountered in the field of biogas upgrading^37−39^) has been calculated:^27^
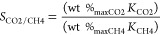
2The wt %_max_ values for both CH_4_ and CO_2_ adsorptions reflect the experimental trends and confirm the differences between the uptakes of hybrid materials and pristine MOFs.

**Table 4:** Degree of Surface Heterogeneity (*t*), Asymptotic Maximum CO_2_ and CH_4_ Adsorption Capacity (wt %_max_), and Equilibrium Constant (*K*) Calculated from Fitting Experimental Adsorption Isotherm to the Töth Equation^a^

	CH_4_			CO_2_			
Sample	wt %_max_	*K*	*t*	wt %_max_	*K*	*t*	*S*_(CO2/CH4)_
HKUST-1^b^	18.98 ± 0.05	0.08 ± 0.01	1.00 ± 0.01	66.2 ± 0.2	0.37 ± 0.01	1.00 ± 0.01	16.14
HKUST-1/GL-ox	14.80 ± 0.09	0.08 ± 0.01	1.00 ± 0.01	50.8 ± 0.2	0.38 ± 0.01	1.00 ± 0.01	16.30
HKUST-1/GL^b^	4.75 ± 0.03	0.23 ± 0.01	1.00 ± 0.01	29.9 ± 0.4	0.31 ± 0.05	0.80 ± 0.01	8.48
HKUST-1/GL-NH_2_	23.60 ± 0.14	0.07 ± 0.01	1.00 ± 0.01	77.6 ± 0.6	0.34 ± 0.10	1.00 ± 0.01	15.97

a Selectivity (*S*) values calculated with eq 2 for a simulated 50:50 CO_2_ and CH_4_ mixture.

b From ref (27).

The values of the *t* parameter, evaluated on the basis of CH_4_ isotherms, are all always equal to 1, and the values calculated on the basis of CO_2_ isotherms are always equal to 1, with the exception of HKUST-1/GL. *t* equal to 1 indicates a very high surface homogeneity, so it can be speculated that the intercalation of the HKUST-1 structure with GL materials induces a structural inhomogeneity that leads to a worsening of the CO_2_ adsorption capacities.

As concerns the values calculated for the adsorption selectivity, for HKUST-1/GL-ox and HKUST-1/GL-NH_2_, a value around 16, comparable to that of the pristine MOF, is calculated, while a very low value (∼8.5) is calculated for HKUST-1/GL, indicating that the introduction of the GL in the Cu-HKUST-1 structure is detrimental for selectivity toward CO_2_.

## Conclusions

In this work, the capacities to capture CO_2_ and store CH_4_ at high pressure by Cu-HKUST-1 based hybrids have been evaluated. The hybrid materials have been prepared by intercalating Cu-HKUST-1 structure with three graphene related materials (GRMs) obtained from the chemical demolition of nanostructured carbon black: oxidized graphene-like (GL-ox) materials, hydrazine reduced graphene-like (GL) materials, and amine-grafted graphene-like (GL-NH_2_) materials. All the hybrid materials showed a good affinity toward both CO_2_ and CH_4_, but the adsorption capacities showed some differences with respect to the pristine material HKUST-1. Indeed, the gas uptakes evaluated for HKUST-1/GL and HKUST-1/GL-ox are overall lower than those of the pristine material (HKUST-1), while better performances were obtained with HKUST-1/GL-NH_2_ for both gases (CO_2_ and CH_4_). The worsening of the gas adsorption capacity compared to that of the pristine MOF suggested that the intercalation with GL or its oxidized form induces changes in the properties/chemistry of the pores that limit the adsorption properties despite the promising textural properties (high surface area, high pore volumes), while the presence of nitrogen containing groups decorating GL materials in HKUST-1/GL-NH_2_ generates a surface chemistry inside the pores more favorable to the adsorption of the analyzed gas (CO_2_ is an acidic gas). The results here are in line with those already reported in the open literature for similar materials (MOF/GRM hybrids) and confirm that the intercalation of the MOF structure with functionalized carbon-based structures promotes the development of additional adsorption sites for CO_2_ adsorption. The presence of reactive sites enables strong interactions and greater selectivity of the adsorption, which are important features in gas purification.

## Supporting Information Available

FTIR spectra of GRMs (Figure S1); enlargement of N_2_ adsorption isotherms at 77 K in the low-pressure range (Figure S2); additional adsorption/desorption isotherms of the investigated materials (Figures S3 and S4) (PDF)

## Abbreviations

BET: Brunauer–Emmett–Teller method
BTC: benzene-1,3,5-tricarboxylic acid
CB: carbon black
CCS: carbon capture and storage
CCUS: carbon capture utilization and storage
DETA: diethyltriamine
DMF: N,N dimethylformamide
DCM: dichloromethane
EDTA: ethylene diamine tetraacetic acid
EDX: energy dispersive X-ray
f-PcT: fast pressure–concentration–temperature
FTIR: Fourier-transform infrared spectroscopy
GL: graphene-like materials
GL-ox: oxidized graphene-like materials
GL-NH_2_: amine functionalized graphene-like materials
G: graphene
GO: graphene oxide
GRM: graphene related material
ICP-MS: inductively coupled plasma-mass spectrometry
MOF: metal organic framework
PCN: porous coordination networks
PSD: pore size distribution
rGO: reduced graphene oxide
RT: room temperature
S: selectivity
SEM: scanning electron microscope
SSA: specific surface area
TGA: thermogravimetric analysis
wt %: maximum storage capacity
wt %_max_: asymptotic maximum storage capacity
XRD: X-ray diffraction
