# Supplementary material for: CO2 Capture and Gas Storage Capacities Enhancement of HKUST-1 by Hybridization with Functionalized Graphene-like Materials
Source: Energy Fuels. 2023 Mar 15;37(7):5291–302. doi: 10.1021/acs.energyfuels.2c04289 (PMC10084447; doi:10.1021/acs.energyfuels.2c04289)
Supplement: Supplementary file 1 — ef2c04289_si_001.pdf [file ef2c04289_si_001.pdf]

Supporting information

CO_2 capture and gas storage capacities enhancement of HKUST-1 by hybridization with functionalized graphene-like materials

Valentina Gargiulo[1], *Alfonso Policicchio*[2,3,4], *Luciana Lisi*[1], *Michela Alfe*[1] *

[1] CNR-STEMS Institute of Sciences and Technologies for Sustainable Energy and Mobility, P. le V. Tecchio 80, 80125 Napoli (NA), Italy.

[2] Dipartimento di Fisica, Università della Calabria, Via P. Bucci - Cubo 31C, 87036 Arcavacata di Rende (CS), Italy

[3] CNISM - Consorzio Nazionale Interuniversitario per le Scienze fisiche della Materia, Via della Vasca Navale 84, 00146 Roma (RM), Italy

[4] Consiglio Nazionale delle Ricerche, Istituto di Nanotecnologia (Nanotec) – UoS Cosenza, Via Ponte P. Bucci, Cubo 31C, 87036 Arcavacata di Rende (CS), Italy.

*Corresponding Author: Michela Alfe, CNR-STEMS, email: michela.alfe@stems.cnr.it

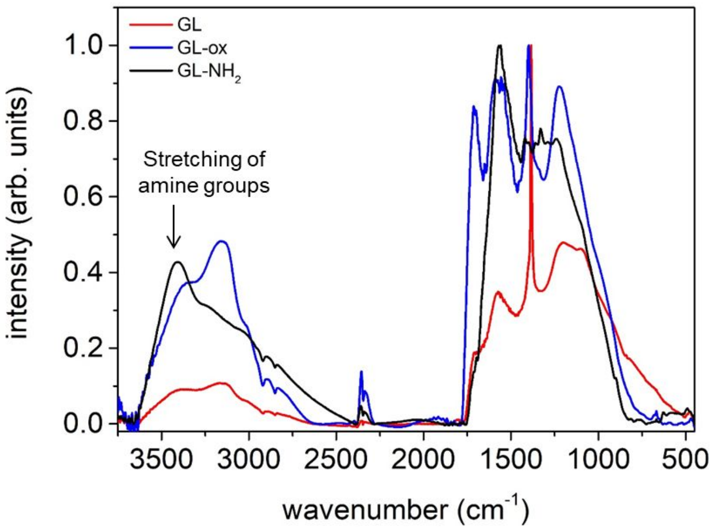

Figure S1. FTIR spectra of the three GRMs in the 450–4000 cm^{-1} region.

The spectrum of the three graphene related materials (GRMs) appear very much alike, and consistent with the broad band appearance typical exhibited by GL materials[1]. The band around 2300 cm^{-1} is ascribable to CO_2 in the environment and it arises as a consequence of the background subtraction. A band attributable to C=O stretching vibrations of the oxygen functional groups (mainly carboxylic groups) deriving from the strong acid top-down demolition treatment is clearly visible in the GL-ox spectrum (1650–1750 cm^{-1}) while a weak shoulder in the GL and GL-NH_2 spectra testifies the presence of residual carbonyl and carboxyl groups in anhydrides, lactones, single ketones and/or quinones. The bands at 1500–1600 cm^{-1} are due to the skeletal vibration of the sp^2 graphitic domains as well as the broad band in the 1300 – 1100 cm^{-1} region is ascribable to the overlapping of multiple sp^2 graphitic skeletal stretching vibrations and of the C-OH and C-O stretching vibrations. Nitrogen atoms in the form of nitro groups were also detected (-NO_2 stretching vibration bands at 1560 and 1350 cm^{-1}) in GL and GL-ox spectra.

The presence of NH_2 functionalities in the GL-NH_2 structure is testified by the prominent band between 3500 and 3350 cm^{-1} in the GL-NH_2 spectrum, standing out the broad band in the 3000–3700 cm^{-1} range related to O-H stretching vibrations (also due to possible adsorbed H_2O) evidenced in GL and GL-ox spectra. The absorption band around 1620 cm^{-1} related to N−H bending mode, is non clearly discernible being submerged by the skeletal vibration of the sp^2 graphitic domains.

[1] a) Alfè, M.; Gargiulo, V.; Di Capua, R.; Chiarella, F.; Rouzaud, J-N.; Vergara, A.; Ciajolo, A. Wet Chemical Method for Making Graphene-like Films from Carbon Black. ACS Appl Mater Interfaces 2012, 4(9), 4491-4498. DOI: 10.1021/am301197q; b) Gargiulo, V.; Alfano, B.; Di Capua, R.; Alfé, M.; Vorokhta, M.; Polichetti, T.; Massera, E.; Miglietta, M. L.; Schiattarella, C.; Di Francia, G. Graphene-like layers as promising chemiresistive sensing material for detection of alcohols at low concentration. J. Appl. Phys. 2018, 123, 024503. DOI: 10.1063/1.5000914; c) Di Capua, R.; Gargiulo, V.; Alfè, M.; De Luca, G. M.; Skála, T.; Mali, G.; Pezzella, A. Eumelanin Graphene-Like Integration: The Impact on Physical Properties and Electrical Conductivity. Front. Chem. 2019, 7, 121. DOI: 10.3389/fchem.2019.00121

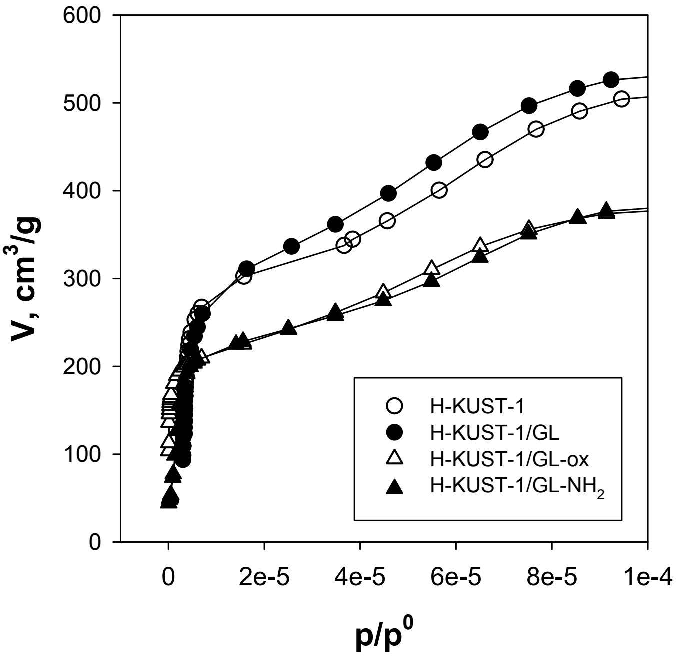

Figure S2. Magnification of N_2 adsorption isotherms at 77 K in the low-pressure range.

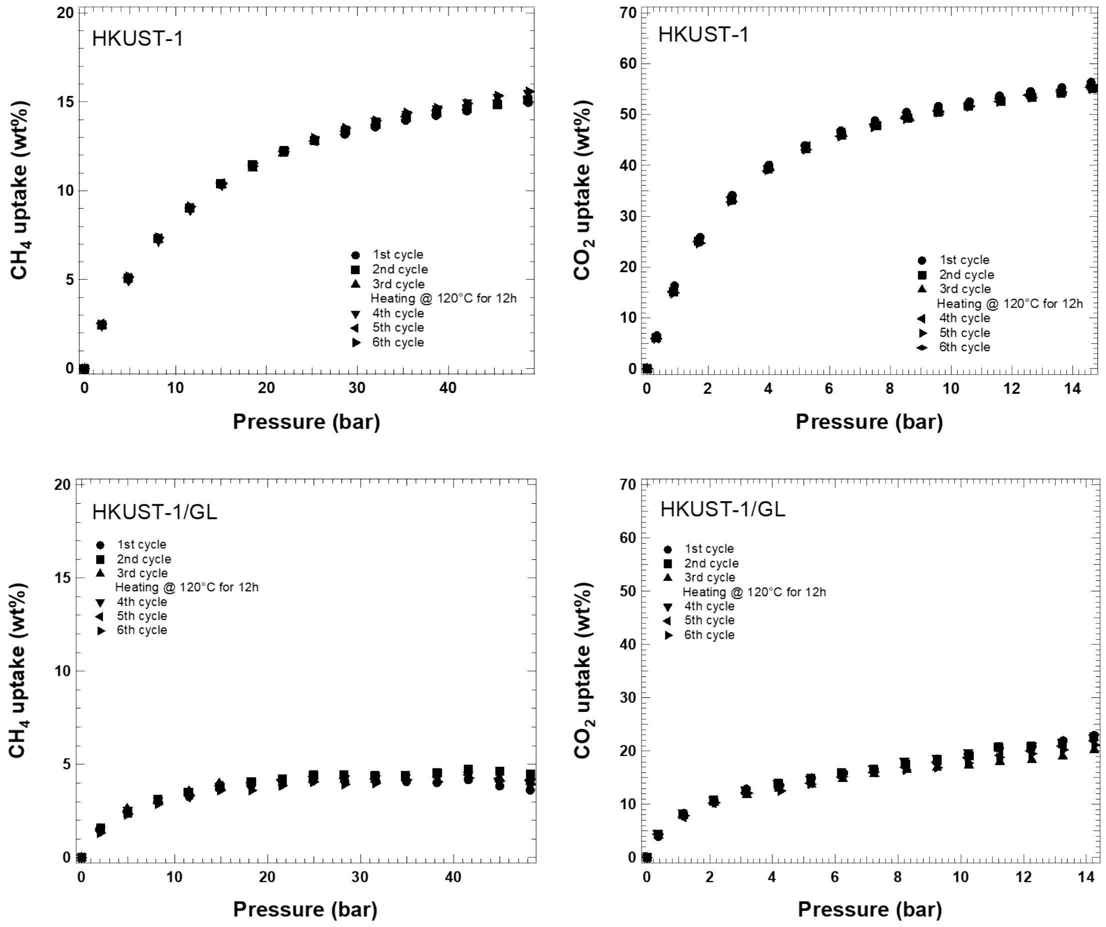

Figure S3. CH_4 adsorption isotherms up to 50 bar and CO_2 adsorption isotherms up to 15 bar of HKUST-1 (up) and HKUST-1/GL-NH_2 (down) recorded during six cycles at RT=23°C. The magnitude of the error is the symbol itself.

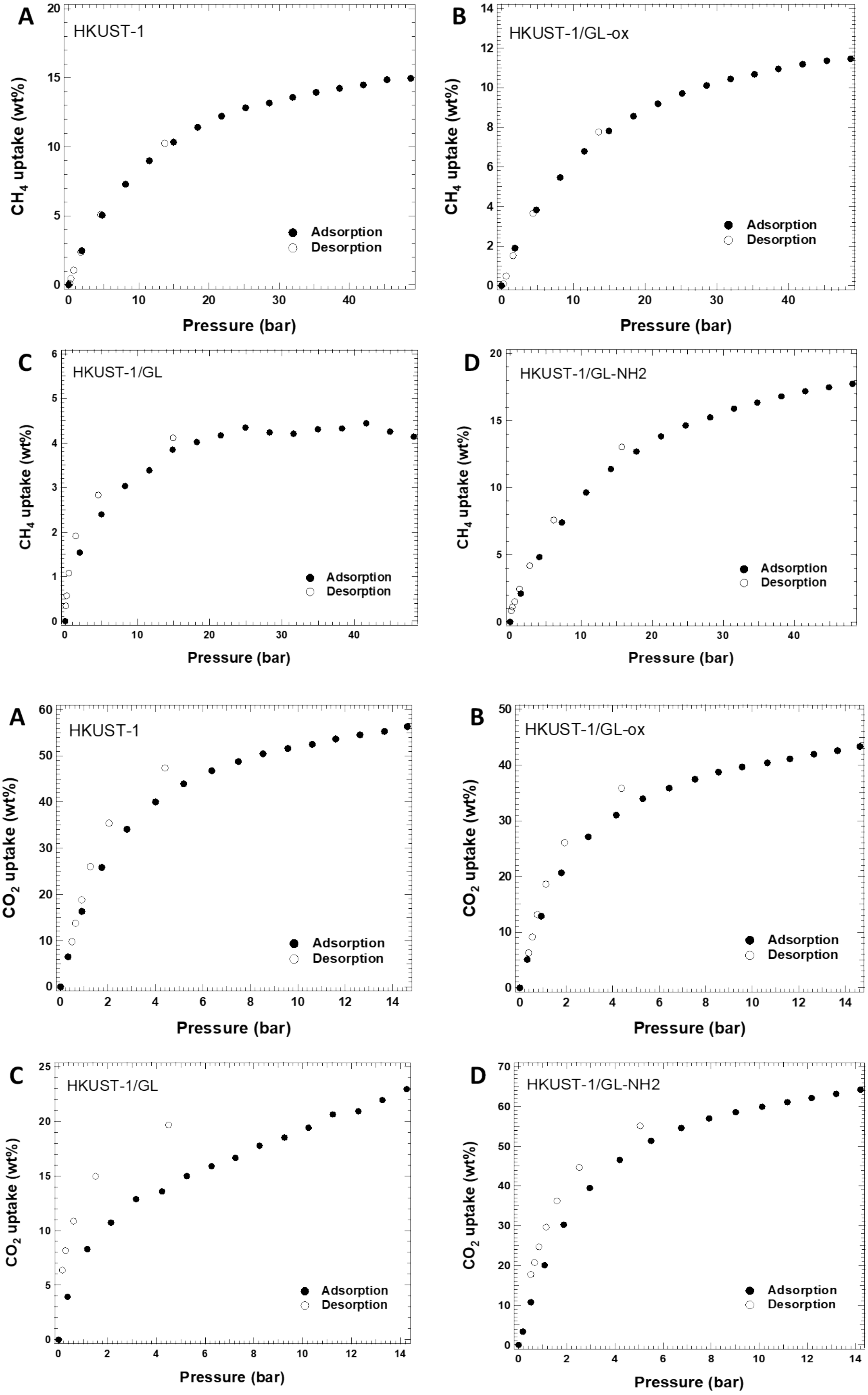

Figure S4. CH_4 and CO_2 adsorption/desorption isotherms (up to 50 bar for CH_4 and up to 15 bar for CO_2) at RT=23°C.
